# Latent Class Analysis of Noninvasive Methods and Liver Biopsy in Chronic Hepatitis C: An Approach without a Gold Standard

**DOI:** 10.1155/2017/8252980

**Published:** 2017-09-13

**Authors:** Flavia F. Fernandes, Hugo Perazzo, Luiz E. Andrade, Alessandra Dellavance, Carlos Terra, Gustavo Pereira, João L. Pereira, Frederico Campos, Maria L. Ferraz, Renata M. Perez

**Affiliations:** ^1^Gastroenterology and Hepatology Department, University of the State of Rio de Janeiro, Rio de Janeiro, RJ, Brazil; ^2^Gastroenterology and Hepatology Department, Bonsucesso Federal Hospital, Rio de Janeiro, RJ, Brazil; ^3^Laboratory of Clinical Research in HIV, AIDS and STD (LAPCLIN-AIDS), National Institute of Infectious Diseases Evandro Chagas (INI), Fundação Oswaldo Cruz (FIOCRUZ), Rio de Janeiro, RJ, Brazil; ^4^Rheumatology Department, Federal University of São Paulo, São Paulo, SP, Brazil; ^5^Department of Research and Development, Fleury Group, São Paulo, SP, Brazil; ^6^Gastroenterology Department, Federal University of São Paulo, São Paulo, SP, Brazil; ^7^D'Or Institute for Research and Education (IDOR), Rio de Janeiro, RJ, Brazil

## Abstract

**Aims:**

To evaluate the applicability of the Latent Class Analysis (LCA) and accuracy of transient elastography (TE), aspartate-to-platelet-ratio-index (APRI), enhanced liver fibrosis (ELF), and liver biopsy (LB) for liver fibrosis assessment in a model without a gold standard.

**Methods:**

Significant fibrosis was defined as TE ≥ 7.1 kPa, APRI ≥ 1.5, ELF ≥ 9.37, or LB METAVIR *F* ≥ 2. Cirrhosis was defined as TE ≥ 12.5 kPa, APRI ≥ 2.0, ELF ≥ 10.31, or LB as METAVIR *F* = 4.

**Results:**

117 patients with chronic hepatitis C were included. In the LCA, for significant fibrosis the sensitivities and specificities (95% CI) were 0.92 (0.86–0.98) and 0.79 (0.72–0.86) for TE; 0.47 (0.40–0.54) and 0.99 (0.95–1.00) for APRI; 0.81 (0.74–0.88) and 0.78 (0.71–0.85) for ELF; and 0.86 (0.68–1.00) and 0.91 (0.79–1.00) for LB. For cirrhosis, the sensitivities and specificities were 0.92 (0.76–1.00) and 0.94 (0.91–0.97) for TE; 0.57 (0.37–0.77) and 0.97 (0.93–1.00) for APRI; 0.94 (0.84–1.00) and 0.88 (0.82–0.94) for ELF; and 0.30 (0.12–0.48) and 1.00 for LB.

**Conclusion:**

LCA was useful to evaluate accuracy of methods for liver fibrosis staging. Sensitivities and specificities of noninvasive methods were increased in LCA compared to the use of LB as the gold standard.

## 1. Introduction

Chronic hepatitis C remains a major public health issue representing one of the leading causes of cirrhosis worldwide [1]. The correct determination of liver fibrosis stage has implications for prognostic, therapeutic, and monitoring purposes [2] and the eradication of hepatitis C virus (HCV) has been associated with lower rates of liver-related complications [3]. Serological biomarkers, such as FibroTest, FibroMeter, aspartate-to-platelet-ratio-index (APRI), and enhanced liver fibrosis (ELF), or imaging methods, such as transient elastography (TE), have been recommended to stage liver fibrosis in chronic hepatitis C [4]. TE has been described as an accurate method for fibrosis assessment in HIV/HCV coinfected patients [5, 6]. Platelet count and liver stiffness measurement by TE has been validated to predict large gastroesophageal varices in patients with compensated advanced chronic liver disease [7]. The high efficacy of direct-acting antiviral drugs (DAAs) has revolutionized the management of patients with chronic hepatitis C. However, detection of compensate cirrhosis leads to screening for hepatocellular carcinoma and might change the choice of therapeutic regimen for HCV eradication [8]. Most of noninvasive methods for fibrosis assessment have been developed and validated using liver biopsy as a reference. However, the diagnostic performance of liver biopsy has been challenged by the length of the liver specimen [9], sampling error [10], and intraobserver variability [11]. Therefore, the diagnostic accuracy of noninvasive methods might have been hampered since liver biopsy might not be a perfect gold standard [12].

Latent Class Analysis (LCA) is a mathematical modeling currently applied in qualitative social research to evaluate the accuracy of tests in the lack of a gold standard [13]. In this methodology, a reference standard is constructed based on the combination of observed and estimated tests results from each patient [14]. A potential limitation might be that this combination might not fit the data, which may be due either to nonindependence between the tests (dependency) or to variability of the disease definition (within-class heterogeneity). In addition, LCA assumes that liver biopsy is binary or dichotomy, whereas fibrosis staging uses a five-stage ordinal scale (from *F*0 to *F*4). So far, this methodology has been used in very few studies in hepatology [12, 15, 16]. The aims of the study were (i) to evaluate the applicability of the LCA models in patients with chronic hepatitis C and (ii) to estimate the sensitivities and specificities of TE, APRI, ELF, and liver biopsy for fibrosis assessment in an approach without a gold standard.

## 2. Material and Methods

### 2.1. Study Design

Patients with chronic hepatitis C from two centers in Rio de Janeiro (University of the State of Rio de Janeiro and Bonsucesso Federal Hospital) were prospective enrolled from April 2011 to July 2012 for this cross-sectional study. Patients with chronic hepatitis C, characterized by the presence of HCV-RNA in blood serum, older than 18 years were included. The exclusion criteria were hepatitis B or human deficiency virus coinfection, self-reported excessive alcohol intake (>40 g/day in men and >20 g/day in women), chronic kidney disease, LB specimens with less than six portal tracts, and unreliable LSM.

Liver fibrosis staging was classified according to the METAVIR scoring [17] and significant fibrosis and cirrhosis were defined as fibrosis stage *F* ≥ 2 and *F* = 4, respectively. Noninvasive tests were performed within 3 months from the date of liver biopsy. The study protocol was conducted in accordance with Helsinki Declaration and was approved by the local ethics committees. All patients signed an informed consent upon enrollment in the study.

### 2.2. Transient Elastography (TE)

TE was performed with M probe of FibroScan® (EchoSens, Paris, France) by an experimented operator (>500 exams) blinded to biomarkers results, following a validated procedure [13]. TE was considered unreliable in presence of any of the following criteria: (i) <10 successful measurements; (ii) an interquartile range (IQR) higher than 30% of the median value; and (iii) a success rate, considered as the ratio between the number of valid and total measures, lower than 60% [18]. Liver stiffness was considered as the median of all valid measurements and fibrosis staging was converted to the METAVIR scoring system as proposed by Castéra et al. [19]: <7.1 as *F*0*F*1; 7.1–9.4 as *F*2; 9.5–12.4 as *F*3; and >12.4 kPa as *F*4.

### 2.3. Serological Biomarkers

Serum alanine aminotransferase (ALT) and aspartate aminotransferase (AST) were measured by enzymatic assay (ADVIA 1200, Siemens, IL, USA). The upper limit of normal (ULN) of aminotransferases values was 55 IU/L and 34 IU/L for ALT and AST, respectively. APRI was calculated according to the following formula: AST level (/ULN)/platelets count (10^9^/L) *∗* 100. Liver fibrosis, estimated by APRI, was converted to the METAVIR scoring system as proposed by Wai et al. [20]: ≥1.5 as *F* ≥ 2 and ≥2.0 as *F* = 4. Calculation of ELF was performed in a frozen serum (−70°C) that was collected in the same day of TE. Serum amino-terminal propeptide of type III procollagen (PIIINP), hyaluronic acid (HA), and tissue inhibitor of matrix metalloproteinases-1 (TIMP-1) were measured in a random access automated clinical immunochemistry analyzer that performs magnetic separation enzyme immunoassay tests (ADVIA Centaur; Siemens Healthcare Diagnostics, Tarrytown, NY). The ELF score was calculated using the algorithm recommended by the manufacturer (Siemens, NY, USA): ELF = 2.278 + 0.851ln⁡(HA) + 0.751ln⁡(PIIINP) + 0.394ln⁡(TIMP-1). Liver fibrosis, estimated by ELF, was converted to the METAVIR scoring system as proposed by Fernandes et al. [21]: >9.37 as *F* ≥ 2 and >10.31 as *F* = 4.

### 2.4. Liver Biopsy

Percutaneous liver biopsies were performed under local anesthesia (FFF) using a 16-G Menghini needle guided by ultrasound as a day-clinic hospitalization. All samples were fixed in a 10% neutral-buffered formalin solution and cut in 5 mm thick sections. Routinely, haematoxylin and eosin, Masson's trichrome, and reticulin stains were performed. Biopsies were classified using the METAVIR scoring system [17] by the same experienced pathologist (FC), who was blinded to clinical characteristics and noninvasive tests results.

### 2.5. Latent Class Analysis (LCA)

LCA is a mathematical modeling that estimates diagnostic accuracy of tests in a scenario where there is no gold standard. The disease status of an individual can be considered as a categorical latent variable such as “disease” or “no disease,” which are named “latent classes.” Through a mathematical method named* standard maximum likelihood,* the modeling aims to obtain a unique solution for constructing a reference standard. Therefore, sensitivities and specificities for each test can be estimated. In addition, the assumption of conditional independence among tests must be respected and data must fit into the model (likelihood ratio goodness-of-fit value [likelihood squared (*L*^2^)] significance >0.05) [22].

In the present study, the LCA models were constructed upon four conditionally independents tests: APRI, ELF, TE, and liver biopsy. APRI and ELF are both serological biomarkers but they include different parameters in the respective formulas. Liver fibrosis is estimated by TE considering the propagation of ultrasound pulses through the hepatic parenchyma and liver biopsy stages fibrosis is based on histological analysis by a semiquantitative score. Two latent class (2LC) models were fitted, one for presence or absence of significant fibrosis (METAVIR *F* ≥ 2) and the other for presence or absence of cirrhosis (METAVIR *F* = 4). In each one of the models, the patient's status could be classified in two mutually exclusive groups: presence or absence of “disease.” Using four tests with a dichotomous result (i.e., positive or negative) in each patient, there were 16 possible combinations for each clinical endpoint. The likelihood of observing each pattern of test results was calculated according to the probability for a positive or negative test. The number of expected cases estimated by LCA models was compared to observed cases for each of the patterns of test results.

### 2.6. Statistical Analysis

Continuous variables were reported as median [interquartile range, IQR] and discrete variables were reported as absolute and relative frequency. Nonparametric tests, Mann–Whitney test for quantitative and Fisher's exact test for qualitative comparisons, were applied. Significance level was determined when *p* ≤ 0.05 assuming two-tailed tests. In the classical 2 × 2 analysis, the performance of TE, APRI, and ELF was assessed using the fibrosis stage obtained by liver biopsy, the classical gold standard. The standard area under the Receiver Operating Characteristics (AUROC) curves for diagnosis of significant fibrosis and cirrhosis was estimated by the empirical (nonparametric) method [23]. In the LCA models, the sensitivities and specificities of each test, including liver biopsy, were assessed without a gold standard. For estimation of tests performance for significant fibrosis and cirrhosis by LCA, the 2LC model that assumed the conditional independence among tests was compared to models with direct effect between tests. The model that better fits for LCA was chosen based on the following criteria: the *p* value of the likelihood squared (*L*^2^) had to be greater than 0.05 and the Bayesian information criterion (BIC) had to be the smallest among all competing models. Statistical analyses were performed using STATA statistical package for Windows (2012; StataCorp LP, College Station, TX, USA) and LEM (log-linear event history analysis with missing data, version 1.0 (Tilburg, Netherlands)).

## 3. Results

Among 131 eligible patients with chronic hepatitis C, 117 patients [34% male gender, median (IQR) age of 55 (48–62) years, and BMI of 26 (24–30) Kg/m^2^] were included. Patients were excluded due to inadequate liver specimen (less than 6 portal tracts) (*n* = 11) or unreliable TE (*n* = 3). Serological biomarkers, APRI and ELF, were reliable in all patients. According to liver biopsy, the prevalence of significant fibrosis and cirrhosis was 46% (*n* = 54) and 7% (*n* = 8), respectively.  Table 1 summarizes clinical and demographic characteristics of included patients.

### 3.1. Classical (2 × 2) Analysis Using Liver Biopsy as a Gold Standard

Using liver biopsy as reference, for diagnosis of significant fibrosis the diagnostic performance [AUROC (95% CI)] of TE, APRI, and ELF was 0.874 (0.811–0.937), 0.810 (0.732–0.887), and 0.807 (0.725–0.889), respectively. In addition, the performance [AUROC (95% CI)] of TE, APRI, and ELF for cirrhosis was 0.942 (0.890–0.993), 0.767 (0.585–0.948), and 0.783 (0.555–1.000), respectively. Sensitivities, specificities, and positive likelihood ratio of noninvasive methods are summarized in Table 2.

### 3.2. Latent Class Analysis without a Gold Standard

The 2LC model that respected the conditional independence among 4 tests (i.e., without direct effect between tests) was the model that better fitted data for LCA. This model presented the lower BIC among the competitive models and a nonsignificant *L*^2^  *p* value for diagnosis of significant fibrosis and cirrhosis (Table 3). The observed and estimated patient's distribution according to the 4 tests results for diagnosis of significant fibrosis and cirrhosis are described in Table 4. The tests were perfectly concordant in 54 (46%) patients (all positive in 20 and all negative in 34 patients) for diagnosis of significant fibrosis and in 77 (66%) patients (all positive in 4 and all negative in 73 patients) for cirrhosis diagnosis.

For diagnosis of significant fibrosis, the sensitivities (95% CI) were 0.92 (0.86–0.98), 0.47 (0.40–0.54), 0.81 (0.74–0.88), and 0.86 (0.68–1.00) for TE, APRI, ELF, and liver biopsy, respectively. In addition, specificities (95% CI) were 0.79 (0.72–0.86), 0.99 (0.95–1.00), 0.78 (0.71–0.85), and 0.91 (0.79–1.00) for TE, APRI, ELF, and liver biopsy, respectively. For cirrhosis, the sensitivities were 0.92 (0.76–1.00), 0.57 (0.37–0.77), 0.94 (0.84–1.00), and 0.30 (0.12–0.48); the specificities were 0.94 (0.91–0.97), 0.97 (0.93–1.00), 0.88 (0.82–0.94), and 1.00 for TE, APRI, ELF, and liver biopsy, respectively (Table 2).

Noninvasive methods performed better when analyzed by LCA in comparison to the classical analysis, using liver biopsy as a reference. For the cirrhosis diagnosis, sensitivity of liver biopsy was reduced and its specificity was similar in LCA compared to classical analysis when liver biopsy was the gold standard (i.e., sensitivity and specificity = 1.00) (Table 2).

## 4. Discussion

This study highlighted the reliability of LCA for assessment of liver fibrosis in a scenario without a gold standard. Sensitivities and specificities of noninvasive methods (APRI, ELF, and TE) were higher in LCA models than when liver biopsy was used as reference. Despite having a satisfactory specificity, the sensitivity of liver biopsy was fair for diagnosis of cirrhosis in LCA model. These results are in concordance with the hypothesis that liver biopsy might not be a perfect gold standard.

Serological biomarkers and new imaging technologies for liver fibrosis assessment have been developed in the last decade [24]. Overall, noninvasive methods for fibrosis staging have been validated using liver biopsy and the reference. However, liver biopsy has been challenged by potential adverse events and sampling error [10, 25]. Therefore, alternative methodologies to validate noninvasive markers for fibrosis assessment without the need of liver biopsy must be implemented and validated. LCA has been described as an accurate mathematical model to evaluate the performance of tests in the absence of gold standard [26].

Using liver biopsy as the reference, we reported an accuracy of TE, APRI, and ELF for significant fibrosis and cirrhosis similar to those described in previous studies [27–29]. Positive and negative likelihood ratios reported in our study were fair and represented a slight effect on posttest probability of presence of significant fibrosis or cirrhosis (Table 2). However, LR+ values were similar to previously published [30] and the low LR− (<0.1) of TE for diagnosis of cirrhosis confirmed that this method might be used for exclusion of this condition in HCV patients. Considering the approach by LCA, the sensitivity of noninvasive methods for diagnosis of significant fibrosis increased when compared to classical analysis (Table 2). Similarly, better results were also reported for TE and FibroTest by authors that used the same mathematical model [12]. In the present study, regardless of the type of methodology used (classical analysis or LCA), TE and APRI were the most sensitive and specific tests for diagnosis of significant fibrosis, respectively (Table 2). In a sensitivity analysis when considering the dual cut-off points proposed for APRI (i.e., ≥0.5 as *F* ≥ 2 and ≥1.0 as *F* = 4) [20], we reported similar results for the accuracy of TE, ELF, and liver biopsy (Supplementary Table 1 in Supplementary Material available online at https://doi.org/10.1155/2017/8252980).

For diagnosis of significant fibrosis, the sensitivity and specificity of liver biopsy decreased in LCA yielding 14% of false negative and 9% of false-positive results. More importantly, we had a substantial decrease in the sensitivity of liver biopsy for cirrhosis diagnosis [0.30 (95% CI 0.12–0.48)]. Despite a decrease in sensitivity, the specificity of liver biopsy to detect significant fibrosis or cirrhosis did not change when LCA was applied. These results were aligned with previous studies: Poynard et al. reported a decrease in sensitivity of liver biopsy from 1.00 (as a gold standard) to 0.51 when LCA was applied [12]. We acknowledge that both studies included different sample size and potential distinct populations. We mostly included female patients with mild fibrosis (54%) and had a low prevalence of cirrhosis (7%). The French study included a more homogenous population, mostly male gender with 15% of cirrhotic patients [12]. In addition, the impact of prevalence of liver fibrosis stages in diagnostic performance of biomarkers based on the spectrum bias might lead to discordance in conclusions for test accuracy [31, 32].

The decrease of sensitivity of liver biopsy in LCA compared to classical analysis might be a reflex of the limitations of liver biopsy. Previous studies reported a considerable discrepancy between liver biopsies performed in both hepatic lobes of the same patient [33], as well as a significant underestimation of severity of liver disease by reduction in the liver specimen length [9]. Bedossa et al. have described that even the “classical 20 mm length” sample could misclassify liver fibrosis in a quarter of patients [10]. Mehta et al. estimated the magnitude of the bias of diagnostic performance of noninvasive methods due to liver biopsy limitations [34].

The diagnostic performance of a noninvasive method might never reach the maximal AUROC value using liver biopsy as the reference due to gold standard limitations [25]. Since noninvasive methods have been validated using histology as the reference, these tests might replicate the false negative and positive of liver biopsy, biasing its diagnostic accuracy. As a practical implication of LCA, the present study confirmed that we must move forward on estimation of diagnostic performance of noninvasive methods for fibrosis staging using methodologies without gold standard.

Noninvasive methods have also important limitations that should be considered. TE performed by the M probe may be unreliable in 20% of patients [35] and this method might have a nonnegligible inter- and intraobserver variability [36, 37]. In addition, liver fibrosis staging by TE might be impacted by presence of necroinflammatory activity, obesity, extrahepatic cholestasis, hepatic congestion, and nonfasting status [38]. In the present study, false-positive rates were higher in obese patients (*n* = 30) compared to those with BMI < 30 Kg/m2 (Supplementary Table 2). ELF includes serum markers involved in the synthesis and breakdown of the extracellular matrix that might be elevated in other systemic diseases not related to liver fibrosis [39]. Finally, APRI has a considerable variability due to laboratory upper limit for normal AST and can be overestimated in presence of necroinflammatory activity due to utilization of transaminases in its formula [40].

We acknowledge that our limited sample (*n* = 117) with a low prevalence of cirrhosis (7%) and single blinded histological analysis were the major limitations of our study. We included treatment-naïve patients with chronic hepatitis C with fibrosis staging for therapeutic decision. The relative low prevalence of cirrhotic patients impacted both methodologies (classical analysis and LCA) to access diagnostic accuracy of noninvasive methods. In addition, Rousselet et al. have demonstrated that a single pathologist specialized in liver histology can accurately stage fibrosis in a liver specimen with a good quality [41]. In the present study, the histological analysis was performed by an experimented liver specialized pathologist (FC) in a specimen with a median of 20 mm and 10 portal tracts. We are aware that LCA models might not be considered per se as a new gold standard for estimation of sensitivities and specificities of noninvasive tests for fibrosis assessment. It should be interpreted as an estimator of accuracy of diagnostic tests with appropriate consideration of limits and strengths [42].

The major strength of this study relies on the fact that we respect the criteria for LCA and that data fitted very well in the model without colinearity between noninvasive tests (2LC model). In our study, latent class rules were strictly respected using four conditionally independent tests into the analysis. In addition, the 2LC model without direct effect between tests was the model that better fitted the data (lower BIC among competitive models and a nonsignificant *L*^2^*p* value) (Table 3). In a sensitivity analysis, similar results for accuracy of the four tests were observed in models with direct effect between noninvasive methods (Supplementary Table 3).

## 5. Conclusion

The application of LCA method was useful to evaluate diagnostic performance of noninvasive methods for liver fibrosis assessment. Sensitivities and specificities of noninvasive methods were increased in LCA compared to the use of liver biopsy as the gold standard. These results reinforced that liver biopsy might be an imperfect reference and that mathematical models without a gold standard should be considered in future studies for validation of noninvasive tests for liver fibrosis staging.

## Supplementary Material

Supplementary Table 1: Performance of tests as estimated by classical 2 x 2 analysis (liver biopsy as gold standard) and Latent Class Analysis (without gold standard) using APRI's cut-off > 0.5 and > 1.0 for diagnosis of significant fibrosis (F≥2) and cirrhosis (F=4), respectively.
Supplementary Table 2: Sensitivities and specificities of tests for diagnosis of significant fibrosis (F≥2) and cirrhosis (F=4) as estimated by Latent Class Analysis in models with co-linearity between non-invasive methods.
Supplementary Table 3: Diagnostic performance of non-invasive tests for diagnosis of significant fibrosis (F≥2) and cirrhosis (F=4) in obese patients (BMI ≥ 30Kg/m2) (n=30).
Supplementary Figure 1: Area under the ROC curve (AUROC) for diagnosis of significant fibrosis (F≥2) of (A) transient elastography (TE), (B) Aspartate-to-Platelet-Ratio-Index (APRI) and (C) Enhanced Liver Fibrosis (ELF) using liver biopsy as the reference.
Supplementary Figure 2: Area under the ROC curve (AUROC) for diagnosis of cirrhosis (F=4) of (A) transient elastography (TE), (B) Aspartate-to-Platelet-Ratio-Index (APRI) and (C) Enhanced Liver Fibrosis (ELF) using liver biopsy as the reference.

## Figures and Tables

**Table 1:** Baseline characteristics of included patients.

	Patients (*n* = 117)
Male gender	40 (34)
Age, years	55 [48–62]
BMI, kg/m^2^	26 [24–30]
ALT, U/L	57 [38–110]
AST, U/L	49 [34–81]
Alkaline phosphatases, U/L	76 [62–99]
GGT, U/L	67 [37–129]
Platelets, ×10^9^/L	212 [174–260]
*Noninvasive methods*	
Transient elastography, kPa	8.3 [6.4–13.6]
*F* ≥ 2, TE ≥ 7.1 kPa	65 (56)
*F* = 4, TE ≥ 12.5 kPa	30 (26)
APRI	0.68 [0.43–1.37]
*F* ≥ 2, APRI ≥ 1.5	27 (23)
*F* = 4, APRI ≥ 2.0	18 (15)
ELF	9.39 [8.70–10.49]
*F* ≥ 2, ELF ≥ 9.37	59 (50)
*F* = 4, ELF ≥ 10.31	36 (31)
*Liver biopsy*	
Specimen length, mm	20 [10–30]
Portal tracts, *n*	10 [8–12]
Fibrosis, METAVIR	
*F*0*F*1	63 (54)
*F*2	35 (30)
*F*3	11 (9)
*F*4	8 (7)

Data expressed as median [interquartile range] or absolute (%). ALT, alanine transaminase; APRI, aspartate-to-platelet-ratio-index; AST, aspartate transaminase; BMI, body mass index; ELF, enhanced liver fibrosis; GGT, gamma-glutamyltransferase; TE, transient elastography. Castéra et al. [19], Wai et al. [20], and Fernandes et al. [21] cut-offs were used for fibrosis staging based on transient elastography, APRI, and ELF, respectively.

**Table 2:** Performance of tests for diagnosis of significant fibrosis (*F* ≥ 2) and cirrhosis (*F* = 4) as estimated by classical 2 × 2 analysis (liver biopsy as gold standard) and Latent Class Analysis (without gold standard) considering the model that better fitted data (2LC).

	Sensitivity (95% CI)		Specificity (95% CI)		Positive LR	Negative LR
	Classical 2 × 2	LCA	Classical 2 × 2	LCA		
	*Significant fibrosis (F ≥ 2)*					
TE	0.87 (0.78–0.96)	0.92 (0.86–0.98)	0.71 (0.60–0.82)	0.79 (0.72–0.86)	3.1	0.2
APRI	0.41 (0.27–0.55)	0.47 (0.40–0.54)	0.92 (0.89–0.95)	0.99 (0.95–1.00)	5.1	0.6
ELF	0.78 (0.67–0.89)	0.81 (0.74–0.88)	0.73 (0.62–0.84)	0.78 (0.71–0.85)	2.9	0.3
Liver biopsy	1.00^*∗*^	0.86 (0.68–1.00)	1.00^*∗*^	0.91 (0.79–1.00)	—	—

	*Cirrhosis (F = 4)*					
TE	1.00	0.92 (0.76–1.00)	0.80 (0.71–0.89)	0.94 (0.91–0.97)	4.5	<0.1
APRI	0.50 (0.16–0.84)	0.57 (0.37–0.77)	0.87 (0.81–0.93)	0.97 (0.93–1.00)	3.9	0.6
ELF	0.88 (0.68–1.00)	0.94 (0.84–1.00)	0.73 (0.64–0.82)	0.88 (0.82–0.94)	3.3	0.2
Liver biopsy	1.00^*∗*^	0.30 (0.12–0.48)	1.00^*∗*^	1.00	—	—

^*∗*^Gold standard by definition. 2LC, two latent class; TE, transient elastography; APRI, aspartate-to-platelet-ratio-index; ELF, enhanced liver fibrosis; CI, confidence interval; LCA, Latent Class Analysis; LR, likelihood ratio; AUROC, area under the receiver operator curve. Positive LR and AUROC were calculated by classical analysis using liver biopsy as gold standard. Models that data better fitted (2LC) for diagnosis of significant fibrosis [*L*^2^ of 9.9504 (*p* value = 0.1268)/Bayesian information criteria = −18.6226] and cirrhosis [*L*^2^ of 5.6494 (*p* value = 0.4636)/Bayesian information criteria = −22.9237] were considered for Latent Class Analysis.

**Table 3:** Competitive comparison of latent classes models for assessment of liver fibrosis by noninvasive methods [TE, APRI, and ELF] and liver biopsy.

Model	Model specification	Significant fibrosis (*F* ≥ 2)		Cirrhosis (*F* = 4)
		*L* ^2^ (*p* value)	BIC	*L* ^2^ (*p* value)
2LC	{X, TE | X, APRI | X, ELF | X, LB | X}	9.9504 (0.1268)	−18.6226	5.6494 (0.4636)
2LC with direct effect between TE and APRI	{X, TE APRI | X, ELF | X, LB | X}	7.9601 (0.0931)	−11.0886	4.7289 (0.3163)
2LC with direct effect between TE and ELF	{X, TE ELF | X, APRI | X, LB | X}	7.9397 (0.0938)	−11.1090	5.4854 (0.2410)
2LC with direct effect between APRI and ELF	{X, TE | X, APRI ELF | X, LB | X}	6.2539 (0.1810)	−12.7948	0.1466 (0.9988)

LC, latent class; *L*^2^, likelihood squared; BIC, Bayesian information criterion; TE, transient elastography; APRI, aspartate-to-platelet-ratio-index; ELF, enhanced liver fibrosis. 2LC was the model that data better fits for estimation of significant fibrosis and cirrhosis.

**Table 4:** Observed and estimated frequencies and standardized residual for 16 combinations estimated by the Latent Class Analysis (LCA) model that better fits data (2LC) for diagnosis of significant fibrosis (*F* ≥ 2)and cirrhosis (*F* = 4).

TE	APRI	ELF	LB	Significant fibrosis (*F* ≥ 2)			Cirrhosis (*F*4)	
				Observed	Estimated	Standardized residual	Observed	Estimated
1	1	1	1	20	17.158	0.686	4	3.973
1	1	1	0	2	2.790	−0.473	10	9.516
1	1	0	1	1	3.969	−1.490	0	0.236
1	1	0	0	2	0.706	1.540	0	0.688
1	0	1	1	18	19.618	−0.365	3	2.956
1	0	1	0	4	5.626	−0.686	7	7.642
1	0	0	1	8	5.347	1.147	1	0.176
1	0	0	0	10	9.787	0.068	5	4.813
0	1	1	1	1	1.429	−0.359	0	0.356
0	1	1	0	1	0.297	1.288	2	1.119
0	1	0	0	0	0.352	−0.594	0	0.021
0	1	0	1	0	0.299	−0.547	2	2.090
0	0	1	1	3	2.503	0.314	0	0.265
0	0	1	0	10	9.579	0.136	10	10.173
0	0	0	1	3	3.625	−0.328	0	0.016
0	0	0	0	34	33.915	0.015	73	72.960

2LC, two latent class; TE, transient elastography; APRI, aspartate-to-platelet-ratio-index; ELF, enhanced liver fibrosis; LB, liver biopsy. 0, negative; 1, positive. Latent Class Analysis considered models that data better fitted for diagnosis of significant fibrosis [*L*^2^ of 9.9504 (*p* value = 0.1268)/Bayesian information criteria = −18.6226] and cirrhosis [*L*^2^ of 5.6494 (*p* value = 0.4636)/Bayesian information criteria = −22.9237].

## References

[1] Petruzziello A., Marigliano S., Loquercio G., Cozzolino A., Cacciapuoti C. (2016). Global epidemiology of hepatitis C virus infection: An up-date of the distribution and circulation of hepatitis C virus genotypes. *World Journal of Gastroenterology*.

[2] de Franchis R. (2015). Expanding consensus in portal hypertension: Report of the Baveno VI Consensus Workshop: Stratifying risk and individualizing care for portal hypertension. *Journal of Hepatology*.

[3] Nahon P., Bourcier V., Layese R. (2017). Eradication of Hepatitis C Virus Infection in Patients With Cirrhosis Reduces Risk of Liver and Non-Liver Complications. *Gastroenterology*.

[4] European Association for Study of Liver (2015). EASL-ALEH Clinical Practice Guidelines: non-invasive tests for evaluation of liver disease severity and prognosis. *Journal of Hepatology*.

[5] Li Vecchi V., Giannitrapani L., Di Carlo P. (2013). Non-invasive assessment of liver steatosis and fibrosis in HIV/HCV- and HCV- infected patients. *Annals of Hepatology*.

[6] Vecchi V. L., Soresi M., Colomba C. (2010). Transient elastography: A non-invasive tool for assessing liver fibrosis in HIV/HCV patients. *World Journal of Gastroenterology*.

[7] Maurice J. B., Brodkin E., Arnold F. (2016). Validation of the Baveno VI criteria to identify low risk cirrhotic patients not requiring endoscopic surveillance for varices. *Journal of Hepatology*.

[8] European Association for the Study of the Liver (2017). EASL Recommendations on Treatment of Hepatitis C 2016. *Journal of Hepatology*.

[9] Colloredo G., Guido M., Sonzogni A., Leandro G. (2003). Impact of liver biopsy size on histological evaluation of chronic viral hepatitis: the smaller the sample, the milder the disease. *Journal of Hepatology*.

[10] Bedossa P., Dargère D., Paradis V. (2003). Sampling variability of liver fibrosis in chronic hepatitis C. *Hepatology*.

[11] Goldin R. D., Goldin J. G., Burt A. D. (1996). Intra-observer and inter-observer variation in the histopathological assessment of chronic viral hepatitis. *Journal of Hepatology*.

[12] Poynard T., De Ledinghen V., Zarski J. P. (2012). Relative performances of FibroTest, Fibroscan, and biopsy for the assessment of the stage of liver fibrosis in patients with chronic hepatitis C: A step toward the truth in the absence of a gold standard. *Journal of Hepatology*.

[13] Mathur C., Stigler M., Lust K., Laska M. (2014). A Latent Class Analysis of Weight-Related Health Behaviors Among 2- and 4-Year College Students and Associated Risk of Obesity. *Health Education and Behavior*.

[14] Qu Y., Tan M., Kutner M. H. (1996). Random effects models in latent class analysis for evaluating accuracy of diagnostic tests. *Biometrics. Journal of the International Biometric Society*.

[15] Poynard T., Munteanu M., Luckina E. (2013). Liver fibrosis evaluation using real-time shear wave elastography: applicability and diagnostic performance using methods without a gold standard. *Journal of Hepatology*.

[16] Yan K., Bridges J. F. P., Augustin S., Laine L., Garcia-Tsao G., Fraenkel L. (2015). Factors impacting physicians' decisions to prevent variceal hemorrhage. *BMC Gastroenterology*.

[17] Bedossa P. (1994). Intraobserver and interobserver variations in liver biopsy interpretation in patients with chronic hepatitis C. *Hepatology*.

[18] Castera L., Goutte N., Tubach F., Bedossa P., Degos F. (2012). How does transient elastography applicability impact on diagnostic accuracy? Results in 1044 patients with viral hepatitis and liver biopsies > 20 mm from a multicenter prospective cohort Fibrostic. *Hepatology*.

[19] Castéra L., Vergniol J., Foucher J. (2005). Prospective comparison of transient elastography, fibrotest, APRI, and liver biopsy for the assessment of fibrosis in chronic hepatitis C. *Gastroenterology*.

[20] Wai C., Greenson J. K., Fontana R. J. (2003). A simple noninvasive index can predict both significant fibrosis and cirrhosis in patients with chronic hepatitis C. *Hepatology*.

[21] Fernandes F. F., Dellavance A., Andrade L. C. (2014). New Cut-off Points of Enhanced Liver Fibrosis (ELF) Panel for Chronic Hepatitis C Patients. *Hepatology*.

[22] Rutjes A. W., Reitsma J. B., Coomarasamy A., Khan K. S., Bossuyt P. M. (2007). Evaluation of diagnostic tests when there is no gold standard. A review of methods. *Health Technology Assessment*.

[23] DeLong E. R., DeLong D. M., Clarke-Pearson D. L. (1988). Comparing the areas under two or more correlated receiver operating characteristic curves: a nonparametric approach. *Biometrics*.

[24] Shi Y., Xia F., Li Q. (2016). Magnetic resonance elastography for the evaluation of liver fibrosis in chronic hepatitis B and C by using both gradient-recalled echo and spin-echo echo planar imaging: a prospective study. *The American Journal of Gastroenterology*.

[25] Bedossa P., Carrat F. (2009). Liver biopsy: the best, not the gold standard. *Journal of Hepatology*.

[26] Green M. J. (2014). Latent class analysis was accurate but sensitive in data simulations. *Journal of Clinical Epidemiology*.

[27] Chou R., Wasson N. (2013). Blood tests to diagnose fibrosis or cirrhosis in patients with chronic hepatitis C virus infection: a systematic review. *Annals of Internal Medicine*.

[28] Bota S., Herkner H., Sporea I. (2013). Meta-analysis: ARFI elastography versus transient elastography for the evaluation of liver fibrosis. *Liver International*.

[29] Xie Q., Zhou X., Huang P., Wei J., Wang W., Zheng S. (2014). The performance of enhanced liver fibrosis (ELF) test for the staging of liver fibrosis: A meta-analysis. *PLoS ONE*.

[30] Castera L., Forns X., Alberti A. (2008). Non-invasive evaluation of liver fibrosis using transient elastography. *Journal of Hepatology*.

[31] Poynard T., de Ledinghen V., Zarski J.-P. (2011). FibroTest and Fibroscan performances revisited in patients with chronic hepatitis C. Impact of the spectrum effect and the applicability rate. *Clinics and Research in Hepatology and Gastroenterology*.

[32] Leeflang M. M. G., Rutjes A. W. S., Reitsma J. B., Hooft L., Bossuyt P. M. M. (2013). Variation of a test's sensitivity and specificity with disease prevalence. *CMAJ*.

[33] Regev A., Berho M., Jeffers L. J. (2002). Sampling error and intraobserver variation in liver biopsy in patients with chronic HCV infection. *The American Journal of Gastroenterology*.

[34] Mehta S. H., Lau B., Afdhal N. H., Thomas D. L. (2009). Exceeding the limits of liver histology markers. *Journal of Hepatology*.

[35] Castéra L., Foucher J., Bernard P. H. (2010). Pitfalls of liver stiffness measurement: a 5-year prospective study of 13,369 examinations. *Hepatology*.

[36] Perazzo H., Fernandes F. F., Gomes A., Terra C., Perez R. M., Figueiredo F. A. F. (2015). Interobserver variability in transient elastography analysis of patients with chronic hepatitis C. *Liver International*.

[37] Perazzo H., Fernandes F. F., Soares J. C. (2016). Learning curve and intra/interobserver agreement of transient elastography in chronic hepatitis C patients with or without HIV co-infection. *Clinics and Research in Hepatology and Gastroenterology*.

[38] Perazzo H., Veloso V. G., Grinsztejn B., Hyde C., Castro R. (2015). Factors that could impact on liver fibrosis staging by transient elastography. *International Journal of Hepatology*.

[39] Yoo E. J., Kim B. K., Kim S. U. (2013). Normal enhanced liver fibrosis (ELF) values in apparently healthy subjects undergoing a health check-up and in living liver donors in South Korea. *Liver International*.

[40] Perazzo H., Pais R., Munteanu M. (2014). Variability in definitions of transaminase upper limit of the normal impacts the APRI performance as a biomarker of fibrosis in patients with chronic hepatitis C: ‘APRI c'est fini?’. *Clinics and Research in Hepatology and Gastroenterology*.

[41] Rousselet M.-C., Michalak S., Dupré F. (2005). Sources of variability in histological scoring of chronic viral hepatitis. *Hepatology*.

[42] Reitsma J. B., Rutjes A. W. S., Khan K. S., Coomarasamy A., Bossuyt P. M. (2009). A review of solutions for diagnostic accuracy studies with an imperfect or missing reference standard. *Journal of Clinical Epidemiology*.

