# Supplementary material for: Latent Class Analysis of Noninvasive Methods and Liver Biopsy in Chronic Hepatitis C: An Approach without a Gold Standard
Source: Biomed Res Int. 2017 Sep 13;2017:8252980. doi: 10.1155/2017/8252980 (PMC5615978; doi:10.1155/2017/8252980)

SUPPLEMENTARY MATERIAL

Latent Class Analysis of non-invasive methods and liver biopsy in chronic hepatitis C: an approach without a gold standard

Supplementary Tables

Supplementary Table 1. Performance of tests as estimated by classical 2 x 2 analysis (liver biopsy as gold standard) and Latent Class Analysis (without gold standard) using APRI's cut-off > 0.5 and > 1.0 for diagnosis of significant fibrosis (F≥2) and cirrhosis (F=4), respectively

Supplementary Table 2. Sensitivities and specificities of tests for diagnosis of significant fibrosis (F≥2) and cirrhosis (F=4) as estimated by Latent Class Analysis in models with co-linearity between non-invasive methods

Supplementary Table 3. Diagnostic performance of non-invasive tests for diagnosis of significant fibrosis (F≥2) and cirrhosis (F=4) in obese patients (BMI ≥ 30Kg/m2) (n=30)

Supplementary Figures

Supplementary Figure 1. Area under the ROC curve (AUROC) for diagnosis of significant fibrosis (F≥2) of (A) transient elastography (TE), (B) Aspartate-to-Platelet-Ratio-Index (APRI) and (C) Enhanced Liver Fibrosis (ELF) using liver biopsy as the reference

Supplementary Figure 2. Area under the ROC curve (AUROC) for diagnosis of cirrhosis (F=4) of (A) transient elastography (TE), (B) Aspartate-to-Platelet-Ratio-Index (APRI) and (C) Enhanced Liver Fibrosis (ELF) using liver biopsy as the reference

Supplementary Table 1. Performance of tests as estimated by classical 2 x 2 analysis (liver biopsy as gold standard) and Latent Class Analysis (without gold standard) using APRI's cut-off > 0.5 and > 1.0 for diagnosis of significant fibrosis (F≥2) and cirrhosis (F=4), respectively.

.

	Sensitivity (95%CI)		Specificity (95% CI)
	**Classical 2 x 2**	**LCA**	**Classical 2 x 2**
**Significant fibrosis (F≥2)**			
**TE**	0.87 (0.78-0.96)	0.92 (0.87-0.97)	0.71 (0.60-0.82)
**APRI**	0.93 (0.86-0.99)	0.97 (0.95-0.99)	0.59 (0.50-0.68)
**ELF**	0.78 (0.67-0.89)	0.81 (0.75-0.87)	0.73 (0.62-0.84)
**Liver biopsy**	1.00*	0.89 (0.83-0.95)	1.00*
**Cirrhosis (F=4**			
**TE**	1.00	0.95 (0.84-0.99)	0.80 (0.71-0.89)
**APRI**	0.71 (0.40-0.99)	0.81 (0.73-0.89)	0.63 (0.54-0.72)
**ELF**	0.88 (0.68-1.00)	0.93 (0.87-0.99)	0.73 (0.64-0.82)
**Liver biopsy**	1.00*	0.30 (0.21-0.39)	1.00*

* gold standard by definition. 2LC, two latent class; TE, transient elastography; APRI, aspartate-to-platelet ratio; ELF, enhanced liver fibrosis; CI, confidence interval; LCA, Latent Class Analysis; LR, likelihood ratio; Positive LR was calculated by classical analysis using liver biopsy as gold standard. Models that data better fitted (2LC) for diagnosis of significant fibrosis [L^2 of 4.5757 (p value = 0.5993) / Bayesian information criteria = -23.9974] and cirrhosis [L^2 of 7.1380 (p value = 0.3083) / Bayesian information criteria = -21.4351] were considered for Latent Class Analysis

Supplementary Table 2. Diagnostic performance of non-invasive tests for diagnosis of significant fibrosis (F≥2) and cirrhosis (F=4) in obese patients (BMI ≥ 30Kg/m2) (n=30)

	AUROC (95%CI)	Sensitivity (95%CI)	Specificity (95% CI)	PPV	NPV
**Significant fibrosis (F≥2)**					
**TE**	0.888 (0.773-0.999)	0.94 (0.82-1.00)	0.57 (0.31-0.83)	0.71	0.89
**APRI**	0.875 (0.747-0.999)	0.63 (0.39-0.86)	0.93 (0.79-1.00)	0.91	0.68
**ELF**	0.790 (0.603-0.977)	0.88 (0.71-1.00)	0.64 (0.39-0.89)	0.74	0.82
**Cirrhosis (F=4)**					
**TE**	0.741 (0.556-0.926)	0.97 (0.77-1.00)	0.63 (0.45-0.82)	0.23	0.99
**APRI**	0.698 (0.315-0.999)	0.33 (0.10-0.87)	0.81 (0.67-0.87)	0.17	0.92
**ELF**	0.543 (0.100-0.999)	0.67 (0.13-1.00)	0.48 (0.29-0.67)	0.13	0.93

AUROC, area under the receiver operator curve; PPV, positive predictive value; negative predictive value; TE, transient elastography; APRI, aspartate-to-platelet ratio; ELF, enhanced liver fibrosis; CI,

Supplementary Table 3. Sensitivities and specificities of tests for diagnosis of significant fibrosis (F≥2) and cirrhosis (F=4) as estimated by Latent Class Analysis in models with co-linearity between non-invasive methods

**Model**	**2LC with direct effect between TE and APRI** **[Sensitivity/Specificity]**	**2LC with direct effect between TE and ELF** **[Sensitivity/Specificity]**	**2LC with direct effect between APRI and ELF** **[Sensitivity/Specificity]**
**Significant fibrosis (F≥2)**			
**TE**	0.92 / 0.75	0.93 / 0.82	0.94 / 0.81
**APRI**	0.46 / 0.96	0.46 / 1.00	0.46 / 0.98
**ELF**	0.84 / 0.78	0.82 / 0.82	0.79 / 0.77
**Liver biopsy**	0.91 / 0.91	0.82 / 0.91	0.86 / 0.92
**Cirrhosis (F4)**			
**TE**	0.94 / 0.94	0.92 / 0.95	1.00 / 0.97
**APRI**	0.59 / 0.97	0.56 / 0.97	0.51 / 0.96
**ELF**	0.94 / 0.88	0.95 / 0.89	0.87 / 0.86
**Liver biopsy**	0.30 / 1.00	0.29 / 1.00	0.29 / 1.00

2LC, two latent classes; TE, transient elastography; APRI, aspartate-to-platelet ratio; ELF, enhanced liver fibrosis

Supplementary Figure 1. Area under the ROC curve (AUROC) for diagnosis of significant fibrosis (F≥2) of (A) transient elastography (TE), (B) Aspartate-to-Platelet-Ratio-Index (APRI) and (C) Enhanced Liver Fibrosis (ELF) using liver biopsy as the reference

A

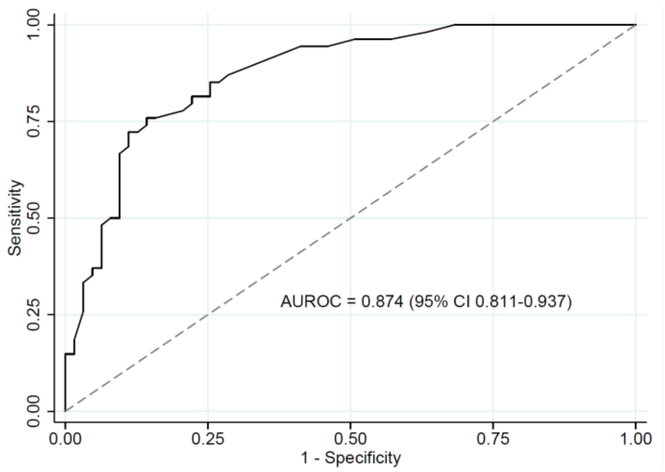

B

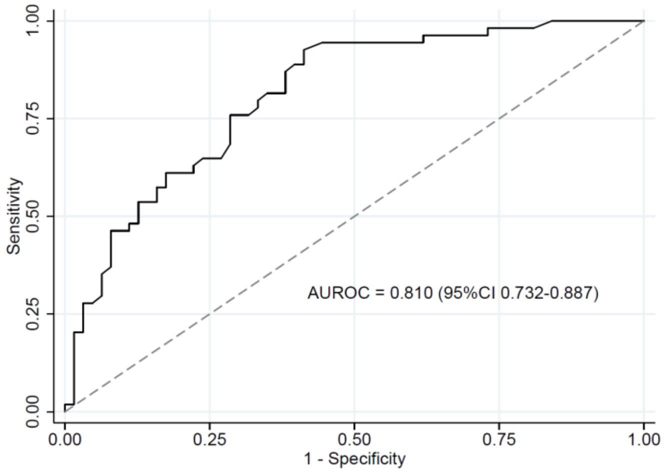

C

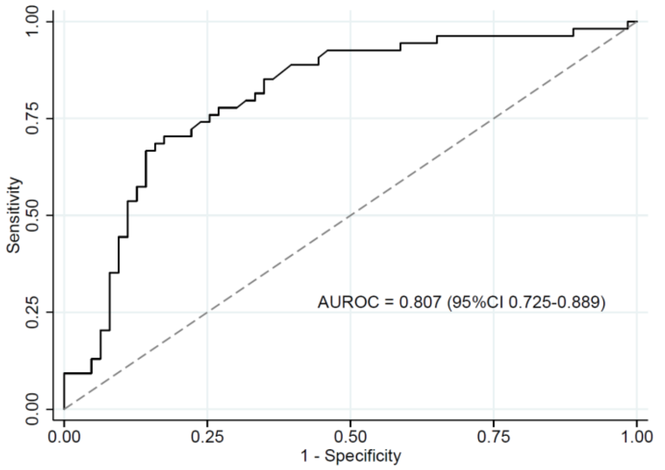

Supplementary Figure 2. Area under the ROC curve (AUROC) for diagnosis of cirrhosis (F=4) of (A) transient elastography (TE), (B) Aspartate-to-Platelet-Ratio-Index (APRI) and (C) Enhanced Liver Fibrosis (ELF) using liver biopsy as the reference

A

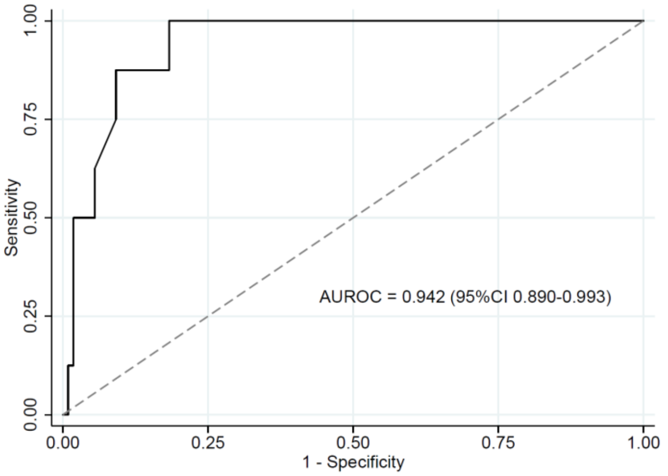

B

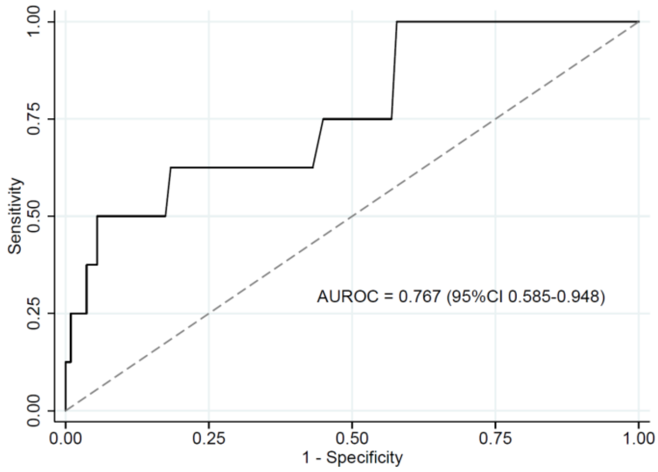

C

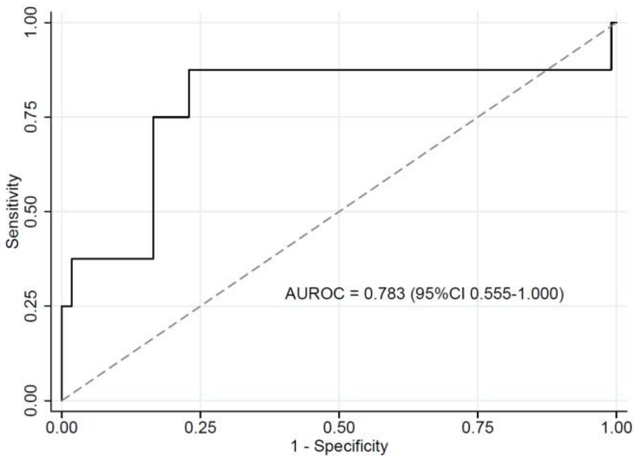

Supplement: Supplementary file 1 — Supplementary Table 1: Performance of tests as estimated by classical 2 x 2 analysis (liver biopsy as gold standard) and Latent Class Analysis (without gold standard) using APRI's cut-off > 0.5 and > 1.0 for diagnosis of significant fibrosis (F≥2) and cirrhosis (F=4), respectively. Supplementary Table 2: Sensitivities and specificities of tests for diagnosis of significant fibrosis (F≥2) and cirrhosis (F=4) as estimated by Latent Class Analysis in models with co-linearity between non-invasive methods. Supplementary Table 3: Diagnostic performance of non-invasive tests for diagnosis of significant fibrosis (F≥2) and cirrhosis (F=4) in obese patients (BMI ≥ 30Kg/m2) (n=30). Supplementary Figure 1: Area under the ROC curve (AUROC) for diagnosis of significant fibrosis (F≥2) of (A) transient elastography (TE), (B) Aspartate-to-Platelet-Ratio-Index (APRI) and (C) Enhanced Liver Fibrosis (ELF) using liver biopsy as the reference. Supplementary Figure 2: Area under the ROC curve (AUROC) for diagnosis of cirrhosis (F=4) of (A) transient elastography (TE), (B) Aspartate-to-Platelet-Ratio-Index (APRI) and (C) Enhanced Liver Fibrosis (ELF) using liver biopsy as the reference. [file 8252980.f1.pdf]
